# Oxypaeoniflorin Ameliorates Titanium Particle‐Stimulated Osteolysis by Targeting Nrf2 to Reprogram Mitochondrial Homeostasis in Osteoclasts

**DOI:** 10.1111/jcmm.71266

**Published:** 2026-07-02

**Authors:** Feng Zhu, Zebin Wu, Jinxuan Hei, Huaqiang Tao, Wenming Li, Kai Chen, Qihan Wang, Dechun Geng, Zhidong Wang, Yaozeng Xu, Shujun Lv

**Affiliations:** ^1^ Department of Orthopedics The First Affiliated Hospital of Soochow University; Orthopedic Institute, Medical College, Soochow University Suzhou Jiangsu China; ^2^ Department of Orthopedics The Chuzhou Hospital Affiliated With Anhui Medical University Chuzhou China; ^3^ Department of Orthopedics Surgery The Affiliated Suzhou Hospital of Nanjing Medical University, Suzhou Municipal Hospital Suzhou Jiangsu Province China; ^4^ Department of Orthopedics Hai'an People's Hospital Jiangsu China

**Keywords:** nrf2, osteoclasts, osteolysis, oxypaeoniflorin, ROS

## Abstract

Wear debris surrounding implants plays a crucial role in the development of periprosthetic osteolysis (PPO) by inducing the aggregation of osteoclasts around the prosthesis. Oxypaeoniflorin (OPF), a derivative of Paeoniflorin, is primarily sourced from the root of the traditional Chinese medicine peony and possesses anti‐inflammatory and antioxidant properties. Currently, the effects of OPF on osteoclast activation and particle‐induced bone resorption remain unclear. In this study, we examined the regulatory effect of OPF on titanium particle‐induced bone erosion in vivo and utilized bone marrow macrophages to investigate the impact of OPF on osteoclast differentiation and resorptive function along with its potential mechanisms. Our results indicate that in vitro, OPF inhibits RANKL‐induced osteoclastogenesis, osteoclast‐dependent resorption, and the expression of osteoclast marker genes through the activation of Nrf2‐mediated oxidative stress signalling. Furthermore, in vivo, OPF suppressed titanium particle‐induced bone loss and osteoclast formation. Our data further confirm the protective effect of OPF against titanium particle‐induced osteolysis from a novel perspective of preventing bone erosion, establishing OPF as a suitable drug for the prevention of PPO.

## 1 Introduction

Total joint replacement (TJA) is a recognized clinical treatment that effectively addresses end‐stage joint diseases resulting from trauma, inflammation, and tumours [1, 2]. The rising trend of population aging is leading to a rapid increase in total joint replacement surgeries. The global annual number of total hip and knee arthroplasties is significantly rising [3]. Periprosthetic osteolysis (PPO) is a major factor contributing to the failure of TJA and the need for revision surgery, and it almost inevitably leads to a decline in patients' quality of life and an increased risk of instability [4].

Wear particles contribute significantly to the gradual bone destruction observed in PPO [5, 6]. Friction during the movement of artificial joints can induce the generation of nanoscale particles derived from the implants, which can stimulate the secretion of various pro‐inflammatory chemokines and cytokines from related cells such as macrophages [7, 8]. These molecules recruit osteoclast precursors and influence osteoblasts and other target cells, enhancing the secretion of receptor activator of nuclear factor kappa‐B ligand (RANKL) and triggering an inflammatory response. This process culminates in the formation of multinucleated mature osteoclasts that adhere to implant surfaces, causing significant bone destruction [9, 10, 11]. Based on the potential pathological mechanisms of PPO, significant efforts have long been devoted to using bone resorption inhibitors for treatment.

Osteoclasts play a special role in the pathological process of PPO, but the underlying mechanisms remain unclear [12]. Osteoclasts originate from bone marrow macrophages (BMMs), with their differentiation mainly driven by macrophage colony‐stimulating factor (M‐CSF) and RANKL [13, 14]. M‐CSF plays a dominant role in enhancing the survival of BMMs, while RANKL promotes the evolution of osteoclast precursor cells [15, 16]. The binding of RANKL to RANK activates key signalling pathways, notably NFATc1, CTSK, and Acp5, which are highly expressed and essential for osteoclast differentiation [17]. Consequently, targeting the specified signals to inhibit bone erosion and osteoclast formation offers a promising approach for treating osteolytic diseases. Interestingly, many agents have been confirmed to modulate the formation and function of osteoclasts, such as bisphosphonates and strontium ranelate, which have been shown to alleviate bone loss caused by wear particles. However, the side effects of these agents include osteonecrosis of the jaw, renal toxicity, and others [18, 19, 20]. Therefore, further exploration for effective strategies against osteoclast differentiation is crucial for the treatment of PPO.

Oxypaeoniflorin (OPF) is a derivative of the core active ingredient of peony, Paeoniflorin [21]. Currently, Oxypaeoniflorin remains in the early stages of research, with its characteristics and mechanisms primarily inferred from those of its parent compound. The structure of Oxypaeoniflorin shares a structural similarity with Paeoniflorin, which has been demonstrated to mitigate AngII‐induced cardiac hypertrophy in H9c2 cells through modulation of oxidative stress and the Nrf2 signalling pathway [22]. Additionally, Paeoniflorin exhibits chondroprotective effects through the regulation of the circ‐PREX1/miR‐140‐3p/WNT5B axis under IL‐1β stimulation [23]. In recent studies, Oxypaeoniflorin has been revealed to improve myocardial ischaemia/reperfusion injury by activating the Sirt1/Foxo1 signalling pathway [24]. Given that the excessive activation of osteoclasts and the progression of PPO involve complex uncontrolled redox effects within the body, it is worth further investigating whether oxypaeoniflorin has an effect on osteoclast activation and the treatment of PPO.

In this study, we investigated the potential protective role of OPF against wear particle‐induced osteolysis. Using a titanium particle‐induced osteolysis model in vivo and RANKL‐stimulated bone marrow‐derived macrophages in vitro, we found that OPF markedly suppressed osteoclast differentiation, osteoclast‐dependent resorptive activity, and the expression of osteoclast marker genes, accompanied by reduced oxidative stress and activation of Nrf2‐associated signalling. Consistently, OPF attenuated titanium particle‐induced bone loss and osteoclast formation in vivo. Together, these findings suggest that OPF mitigates particle‐driven osteolysis and support modulation of oxidative stress responses as a potential strategy for preventing periprosthetic osteolysis after arthroplasty.

## 2 Materials and Methods

### 2.1 Materials and Drugs

Oxypaeoniflorin was purchased from MedChemExpress. Titanium particles were sourced from Alfa Aesar, Ward Hill, MA. Mouse RANKL and M‐CSF were sourced from Amizona Scientific LLC, China. Dulbecco's modified Eagle's medium (DMEM/high glucose) was obtained from VivaCell in Shanghai, China, and fetal bovine serum (FBS) was procured from HyClone in Logan, USA. TRAcP kit was obtained from Amizona Scientific LLC, CHINA. DAPI solution (5 μg/mL; Cat. No. C0066) was purchased from Beijing Solarbio Science & Technology Co. Ltd. (Solarbio; Beijing, China).

### 2.2 CCK‐8 Detection

We conducted a cell viability assay to evaluate the toxic effects of OPF on BMMs cells, using CCK‐8 (NCM Biotech, China) as the reagent. BMMs were cultured in 96‐well plates and exposed to different OPF concentrations for durations of 1 and 3 days. The cells were then incubated at 37°C for 2 h in α‐MEM medium containing 10% CCK‐8 solution. The absorbance at 450 nm was measured using a microplate reader from BioTek, USA.

### 2.3 Cell Culture and Osteoclast Induction Differentiation

Bone marrow cells were isolated from the femurs and tibias of 8‐week‐old female C57BL/6 mice to obtain primary BMMs. These cells were cultured in α‐MEM medium supplemented with 10% fetal bovine serum, 1% penicillin/streptomycin, and 50 ng/mL M‐CSF. After 24 h, non‐adherent cells were discarded, and the remaining adherent cells were further cultured for 3 days. Subsequently, BMMs were seeded at a density of 9 × 10^4^ cells per well in a 24‐well plate, followed by stimulation with RANKL (50 ng/mL) and M‐CSF (50 ng/mL). After a 4‐day culture period, TRAcP staining was performed using a TRAcP kit, and cells with more than three nuclei were considered mature osteoclasts.

### 2.4 Bone Resorption Experiment

The bone resorption assay was employed to assess bone resorption functionality. BMMs were re‐seeded onto bovine bone slices (Amizona Scientific LLC) and subsequently induced with 50 ng/mL M‐CSF and RANKL, while applying various additional interventions. Cells were extracted from bovine bone slices using ultrasound after 7 days. After a modified gradient ethanol dehydration process, the bone slices were fully dried via critical point drying. The bone slices were then gold‐coated. Bone slice resorption pits were examined with an FEI Quanta 250 scanning electron microscope and quantified using ImageJ software.

### 2.5 Western Blot

Samples were washed twice with PBS solution and lysed in RIPA buffer at 4°C for 30 min before Western blotting. Centrifugation for 25 min produced a protein solution in the supernatant. Protein levels were measured using a BCA assay kit. Proteins were equally loaded, separated using SDS‐PAGE, and transferred onto a nitrocellulose membrane. The membrane was incubated at 4°C for 12 h with primary antibodies, including CTSK (ab187647, Abcam), NFATc1 (ab25916, Abcam), Acp5 (ab185716, Abcam), RhoA (ab187027, Abcam), DC‐STAMP (A14630, Abconal), Nrf2 (16396–1‐AP, Proteintech), HO‐1 (10701–1‐AP, Proteintech), NQO1 (67240–1‐lg, Proteintech), and β‐actin (AC038, Abconal). The membrane underwent three washes with washing solution before a 60‐min incubation with horseradish peroxidase‐conjugated secondary antibody solution. Enhanced chemiluminescence was used to detect relative density, and ImageJ software was employed for quantification of protein bands.

### 2.6 RT‐qPCR

TRIzol reagent was used to extract total RNA from various samples. The concentration of RNA was measured using a NanoDrop 2000 spectrophotometer. One microgram of isolated RNA was reverse transcribed into complementary DNA (cDNA) according to the manufacturer's protocol. Real‐time PCR was conducted using synthesized cDNA as a template, with each cDNA sample combined with specific primers to quantify target gene expression. Table 1 lists the primer sequences utilized in this study. Real‐time PCR was conducted on a CFX96 thermal cycler utilizing an eight‐tube amplification reaction. Each sample was tested in triplicate using *Gapdh* as the reference gene, and fold changes were determined via the 2^−ΔΔCq^ relative quantification method.

**TABLE 1:** Primer sequences for RT‐qPCR.

Gene	Primer sequence (F)	Primer sequence (R)
*Ctsk*	CTTCCAATACGTGCAGCAGA	TCTTCAGGGCTTTCTCGTTC
*Mmp9*	CGTGTCTGGAGATTCGACTTGA	TTGGAAACTCACACGCCAGA
*Nfatc1* *Dc‐stamp* *Oscar* *Nfe2l2* *Hmox1* *Nqo1* *Sod1* *Sod2* *RhoA*	GAGAATCGAGATCACCTCCTAC AAAACCCTTGGGCTGTTCTT TCATCGTGGCAACCATGAAC GGAGTAGGATGAATGAGACAGG GGAAATCATCCCTTGCACGC TCCAGACTCCGATCATCAAGC GCTGTACCAGTGCAGGACCTCAT CAGACCTGCCTTACGACTATGG CAGGCTGTGTTGGCAATAGTG	TTGCAGCTAGGAAGTACGTCTT AATCATGGACGACTCCTTGG GCCTGCTTCTTGATGTCCTC CTTGGCTACTTCAGAATAGCTG TGTTTGAACTTGGTGGGGCT GCTCATGGTGTTCAGAATTGTGT CTCTCCTGAGAGTGAGATCACACGA CTCGGTGGCGTTGAGATTGTT CGGCTATCTCTCTGCTGGAT
*Gapdh*	GGTTGTCTCCTGCGACTTCA	TGGTCCAGGGTTTCTTACTCC

### 2.7 F‐Actin Staining

After the formation of mature osteoclasts, F‐actin ring staining was performed. In brief, cells were fixed with 4% paraformaldehyde, followed by staining with phalloidin (1:200, Yeasen, China) to label F‐actin, and subsequently, the nuclei were stained with DAPI. Finally, images were captured using a fluorescence microscope (Zeiss, Germany).

### 2.8 Immunofluorescence Staining

BMMs were plated on coverslips in 24‐well plates at a density of 9 × 10^4^ cells per well. Various interventions were then applied to induce osteoclast differentiation. Cells on coverslips were fixed with 4% paraformaldehyde, permeabilized using 0.2% Triton X‐100, and blocked for 1 h at 4°C after 4 days. Primary antibodies against ACP5 (ab185716, Abcam) and CTSK (ab187647, Abcam) were then added and incubated overnight at 4°C. Subsequently, the cells were rinsed with PBS, treated with Alexa Fluor 488 secondary antibody, and mounted using a DAPI‐containing fluorescence medium. A Zeiss fluorescence microscope was used for imaging.

### 2.9 RNA Sequencing

BMMs were plated in 6‐well plates at a concentration of 5 × 10^5^ cells per well. The RANKL group received treatment with 50 ng/mL RANKL only, while the RANKL + OPF group was supplemented with 10 μM OPF in addition to RANKL intervention. Total RNA was extracted with TRIzol reagent following a three‐days intervention. The extracted RNA was sequenced by GENEWIZ (Suzhou, China). After quality control, clean reads were used for downstream analysis. Gene expression levels were quantified and normalized, and principal component analysis (PCA), differential expression analysis, and downstream visualization were performed in R. Differentially expressed genes (DEGs) between the two groups were identified using the criteria of |Log_2_ fold change| > 1 and false discovery rate (FDR) < 0.05, with FDR controlled by the Benjamini–Hochberg method. GO and KEGG enrichment analyses were subsequently performed based on the identified DEGs.

### 2.10 Detection of ROS

The generation of ROS was assessed using the 2,7‐dichlorodihydrofluorescein diacetate (DCFH‐DA) fluorescence staining kit. Cells from different groups were added to DCFH‐DA culture medium without fetal bovine serum. After 30 min of incubation, the medium was removed. The cells underwent three PBS washes. The cells were then fixed and observed under a fluorescence microscope, followed by quantitative analysis using ImageJ software. Simultaneously, cells were harvested for flow cytometry detection.

### 2.11 Detection of mtROS

To investigate the effect of OPF on mitochondrial superoxide levels during osteoclast differentiation, this study intervened in osteoclast differentiation with different concentrations of OPF. The mitochondrial superoxide was detected using the MitoSOX Red fluorescent probe, which specifically targets mitochondria and emits red fluorescence upon oxidation by superoxide. The MitoSOX Red stock solution was diluted in a 1:1000 ratio in pre‐warmed serum‐free medium and incubated in the dark for 30 min. After incubation, the cells were washed three times with pre‐chilled PBS buffer to remove unreacted probes. For subcellular localization observation, images were acquired using a Zeiss LSM 880 confocal microscope. Furthermore, cells were harvested for flow cytometric analysis.

### 2.12 Molecular Docking

Compounds in SDF format were sourced from the PubChem database and imported into ChemDraw 3D for energy minimization using the MM2 module, resulting in the lowest energy conformation, which was saved as a mol2 file. The protein structure was downloaded from the UniProt database and visualized using PyMOL. Using MGLTools 1.5.6, the ligand and receptor underwent desolvation, hydrogen addition, charge calculation, and non‐polar hydrogen merging, and were subsequently saved as pdbqt files. AutoDock Vina 1.1.2 was utilized for molecular docking.

### 2.13 Molecular Dynamics (MD)

Molecular dynamics simulations were carried out using GROMACS 2022. The protein topology was generated by pdb2gmx with the AMBER14SB force field, while the ligand parameters were produced using sobtop_1.0 (dev3.1) based on GAFF2 and RESP charges. The complex was solvated in a TIP3P water box with a 1 nm margin, and Na^+^/Cl^−^ ions were added to neutralize the system with an ionic strength of 0.15 M. Long‐range electrostatics were treated using the particle mesh Ewald (PME) method with a 1 nm cutoff, and bonds were constrained using the LINCS algorithm. The system was energy‐minimized (3000 steps of steepest descent followed by 2000 steps of conjugate gradient) and subsequently simulated for 100 ns under the NPT ensemble (310 K, Nosé–Hoover thermostat; 1 bar, Parrinello–Rahman barostat) with a 2 fs time step. Trajectories were analysed by calculating RMSD, RMSF, radius of gyration (Rg), solvent‐accessible surface area (SASA), hydrogen bonds, and free energy landscape, and the MM‐PBSA binding free energy was estimated using the g_mmpbsa package.

### 2.14 Cycloheximide Chase Assay

Cycloheximide (CHX) chase assay was performed to evaluate the protein stability of Nrf2. Briefly, BMMs were treated with RANKL in the presence or absence of OPF as described above. Cells were then incubated with cycloheximide (CHX, 50 μg/mL) to block new protein synthesis and harvested at the indicated time points (0, 20, 40, and 60 min). Total cellular proteins were extracted and subjected to western blot analysis. The relative protein level of Nrf2 was normalized to β‐actin, and the degradation curve was generated based on the normalized Nrf2 intensity at each time point.

### 2.15 Cellular Thermal Shift Assay

Cellular thermal shift assay (CETSA) was performed to assess the direct interaction between OPF and Nrf2 in cells. BMMs were treated with OPF or DMSO control, collected, and resuspended in PBS supplemented with protease inhibitors. The cell suspensions were aliquoted and heated at the indicated temperatures ranging from 37°C to 52°C for 3 min, followed by cooling at room temperature. After repeated freeze–thaw lysis, the samples were centrifuged at 12,000 × g for 15 min at 4°C, and the soluble protein fractions were collected for western blot analysis. The thermal stability of Nrf2 was evaluated by comparing the remaining soluble Nrf2 protein levels between the OPF‐treated and control groups.

### 2.16 Immunofluorescence Analysis of Nrf2 Nuclear Translocation

Immunofluorescence staining was performed to assess the subcellular localization of Nrf2. BMMs were seeded on glass coverslips and treated with RANKL in the presence or absence of different concentrations of OPF. After treatment, cells were fixed with 4% paraformaldehyde, permeabilized with Triton X‐100‐containing permeabilization buffer, and blocked with bovine serum albumin. The cells were then incubated with a primary antibody against Nrf2 overnight at 4°C, followed by incubation with a fluorescent secondary antibody. Nuclei were counterstained with DAPI. Images were captured using a fluorescence microscope, and the nuclear‐to‐cytoplasmic fluorescence intensity ratio of Nrf2 was quantified using ImageJ software.

### 2.17 Titanium Particle‐Induced Calvarial Osteolysis Model

All animal experiments were approved by the Ethics Committee of Soochow University. Twenty‐one 8‐week‐old male C57BL/6 mice were randomly assigned to three groups (*n* = 7 per group): sham surgery with PBS treatment (Sham group), titanium particle implantation with PBS treatment (TiPs group), and titanium particle implantation with 1 mg/kg OPF treatment (TiPs + OPF group). Titanium (Ti) particles (manufacturer‐specified average diameter < 5 μm; Alfa Aesar, Ward Hill, MA, USA) were pretreated to reduce potential endotoxin contamination by baking at 180°C for 6 h, followed by three sequential washes with 70% ethanol and sterile PBS before use. To establish the calvarial osteolysis model, mice were anaesthetised, and the calvarial skin was shaved, disinfected, and incised along the midline. The periosteum was carefully separated from the calvaria, and 40 mg of pretreated Ti particles were uniformly applied to the calvarial surface before the incision was sutured. Mice in the Sham group underwent the same surgical procedure without Ti particle implantation. Daily subperiosteal injections of the respective solutions were administered at the center of the calvaria for 14 consecutive days. All mice were euthanized 14 days after surgery, and the calvaria were harvested and fixed in 4% paraformaldehyde for subsequent analysis.

### 2.18 Micro‐CT Analysis

After fixing the skull, titanium particles were removed from the surface of the calcified area to reduce metal artefacts. Scanning was conducted using a Skyscan 1176 micro‐CT device (Aartselaar, Belgium). NRecon software (Skyscan micro‐CT, Aartselaar, Belgium) was used to reconstruct 2D and 3D images. Bone parameters such as bone mineral density (BMD, mg/cm^3^), bone volume (BV, mm^3^), bone volume to tissue volume ratio (BV/TV, %), trabecular number (Tb.N, 1/mm), and total porosity (%) were analysed using Skyscan software.

### 2.19 Histological Staining

Cranial samples were decalcified in 10% EDTA solution, then embedded in paraffin and prepared into tissue sections. After deparaffinization, paraffin sections were subjected to a gradient rehydration process through a decreasing concentration ethanol series, with each stage lasting 2 min, facilitating the gradual restoration of the tissue to a physiological hydration state. These sections were first deparaffinized with xylene, followed by gradient hydration and stained with haematoxylin–eosin as well as TRAcP staining. Section images were captured using an Axiovert 40C optical microscope (Zeiss, Germany).

### 2.20 Statistical Analysis

Data are presented as mean ± standard deviation (SD). Statistical analyses were performed using GraphPad Prism 8.0. For in vivo experiments, each mouse was considered one biological replicate. For in vitro experiments, independently repeated experiments were considered biological replicates, whereas repeated wells or measurements within the same experiment were considered technical replicates. Comparisons between two groups were performed using the unpaired two‐tailed Student's *t*‐test. Comparisons among three or more groups were analysed using one‐way ANOVA followed by Tukey's multiple comparisons test. Sample sizes (n) are indicated in the corresponding figure legends unless otherwise specified. A value of *p* < 0.05 was considered statistically significant.

## 3 Results

### 3.1 OPF Inhibits Osteoclast Activation and Bone Resorption in a Dose‐Dependent Manner

**FIGURE 1:**
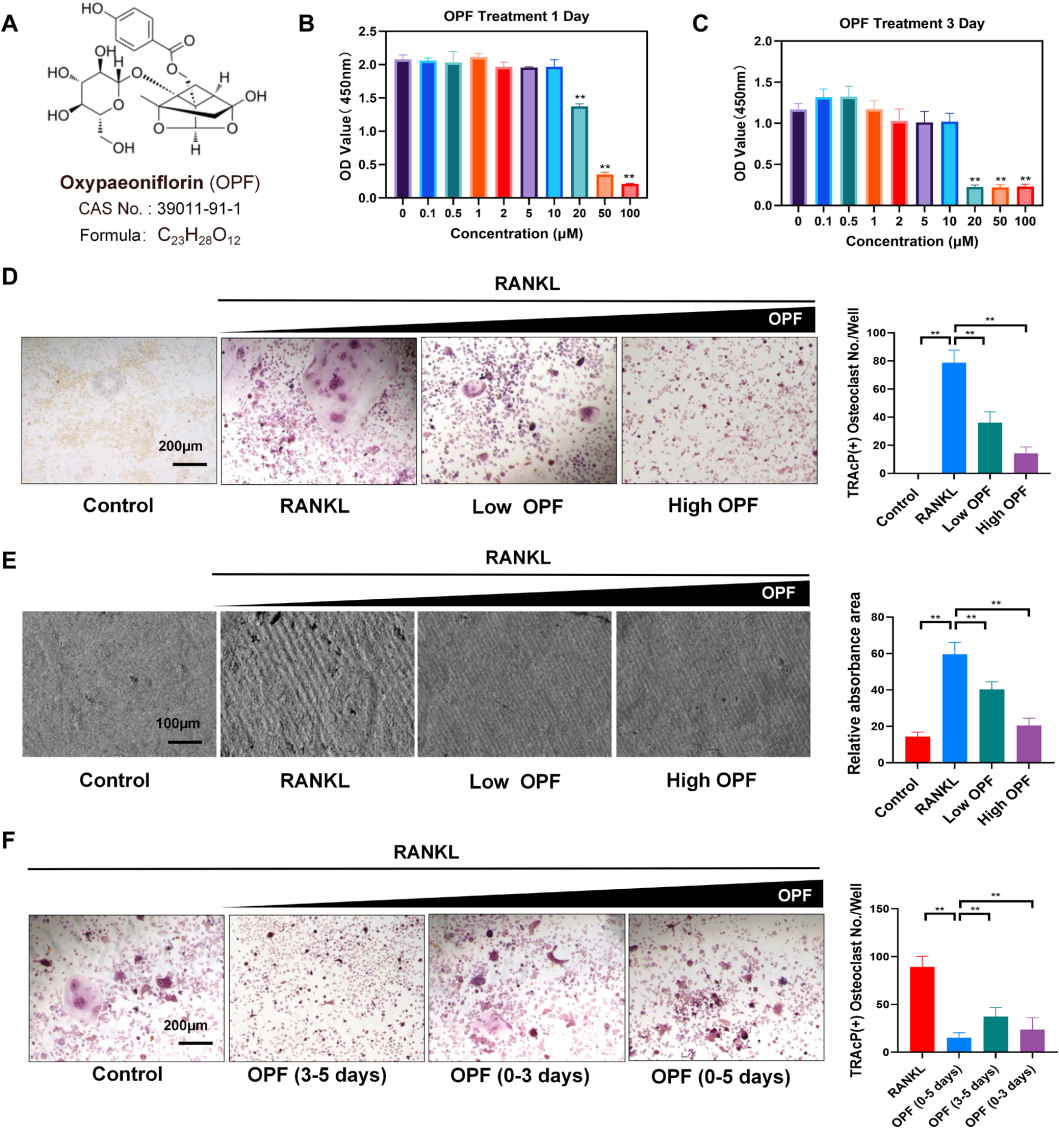
OPF inhibits osteoclast activation and bone resorption in a dose‐dependent manner. (A) Structural formula of OPF. (B) CCK8 assay reveals the cytotoxicity of OPF intervention on BMMs after 1 day, *n* = 3. (C) CCK8 assay reveals the cytotoxicity of OPF intervention on BMMs after 3 days, *n* = 3. (D) TRAcP staining shows the effect of OPF intervention on osteoclast differentiation, *n* = 3, Scale bar = 200 μm. (E) Bone resorption experiment reveals the impact of OPF intervention on osteoclast bone resorption, *n* = 3, Scale bar = 100 μm. **p < 0.01 relative to RANKL group. (F) TRAcP indicates the effect of OPF intervention on osteoclast differentiation at different time points, *n* = 3, Scale bar = 200 μm.

The chemical structure of OPF is shown in Figure 1A. The cytotoxic effects of OPF on BMMs were assessed using the CCK‐8 assay at 24 and 72 h. The results indicated that at concentrations below 10 μM, the effect on cell viability was minimal compared to the control group. Therefore, in this study, 5 μM was chosen as the low concentration intervention group, and 10 μM as the high concentration intervention group (Figure 1B,C). To explore the effects of OPF on osteoclast generation, high and low concentrations of OPF were used to intervene in RANKL‐induced osteoclasts. The addition of 50 ng/mL RANKL induced the formation of TRAcP‐positive osteoclasts; however, OPF significantly inhibited osteoclast formation in a dose‐dependent manner (Figure 1D) and significantly suppressed the trend of increased bone resorption caused by osteoclasts on the bone surface following RANKL treatment, and the inhibitory effect of OPF on the resorptive capacity of osteoclasts was gradually enhanced with increasing concentrations (Figure 1E). Furthermore, this inhibitory effect was most pronounced in the early intervention period (1–3 days) (Figure 1F). Taken together, our results indicate that OPF inhibits osteoclastogenesis and bone resorption in a dose‐dependent manner.

### 3.2 OPF Inhibits the Expression of Osteoclast‐Related Markers

**FIGURE 2:**
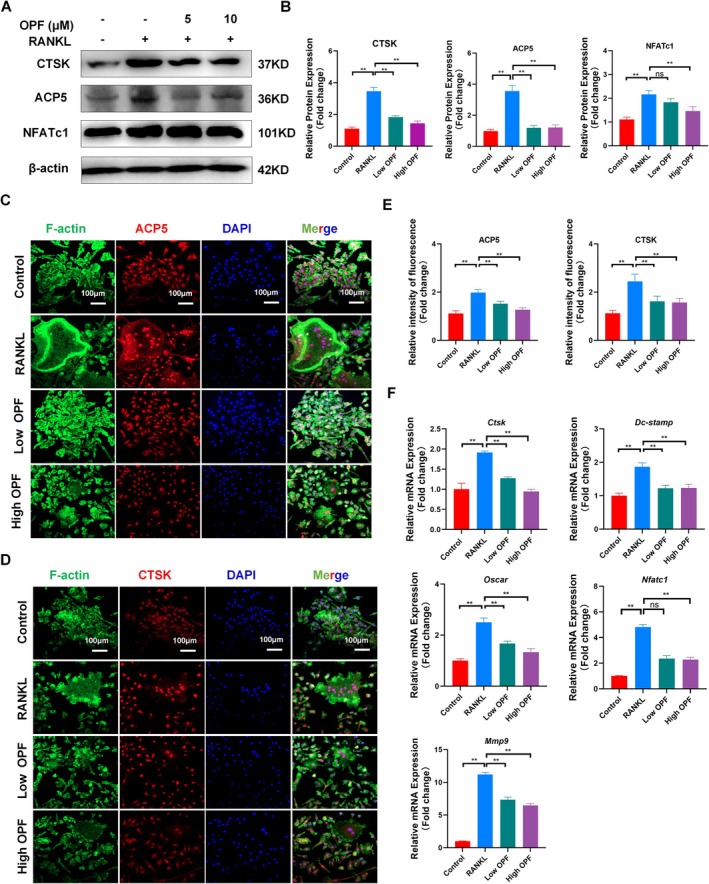
OPF inhibits the expression of osteoclast‐related markers. (A) Western blot analysis reveals the effects of OPF intervention on the expression of CTSK, ACP5, and NFATc1, *n* = 3. (B) Quantitative analysis of CTSK, ACP5, and NFATc1 proteins, *n* = 3. (C) Immunofluorescence co‐staining of ACP5 (red) and F‐Actin (green) in osteoclasts, *n* = 3, Scale bar = 100 μm. (D) Immunofluorescence co‐staining of CTSK (red) and F‐Actin (green) in osteoclasts, *n* = 3, Scale bar = 100 μm. (E) Quantitative immunofluorescence analysis of ACP5 and CTSK. (F) RT‐PCR shows the changes in the expression of osteoclast‐related marker genes *Ctsk*, *Mmp9*, *Nfatc1*, *Dc‐stamp*, and *Oscar* following OPF intervention, *n* = 3. **p < 0.01 relative to RANKL group.

NFATc1 is a key regulatory factor in osteoclast differentiation, which activates genes related to osteoclast differentiation upon stimulation by RANKL signalling [25]. CTSK, as an effector molecule, is responsible for the degradation of bone matrix [26]. MMP9 is also regulated by NFATc1 and is involved in the remodelling of the extracellular matrix, promoting osteoclast migration and bone resorption [27]. Our Western blot results reveal that after RANKL intervention induces osteoclast differentiation, a concentration gradient of OPF significantly inhibits the expression of these proteins (Figure 2A,B). Immunofluorescence staining results suggest that after RANKL intervention, there is an increase in osteoclast formation, along with elevated intracellular levels of CTSK and ACP5. Conversely, following OPF intervention, there is a significant inhibition of osteoclast formation, with a marked reduction in intracellular CTSK and ACP5 expression (Figure 2C,E). OSCAR primarily activates relevant signalling pathways such as NFATc1 in the early stages of osteoclast differentiation, while Dc‐stamp regulates the fusion process of osteoclast precursors, which is a prerequisite for the formation of multinucleated giant cells [28, 29]. Our RT‐PCR results indicate that OPF not only downregulates the expression of *Mmp9*, *Ctsk*, and *Nfatc1* but also significantly inhibits the expression of *Oscar* and *Dc‐stamp* (Figure 2F).

### 3.3 OPF Involvement in Regulating Osteoclast Cytoskeleton Formation

**FIGURE 3:**
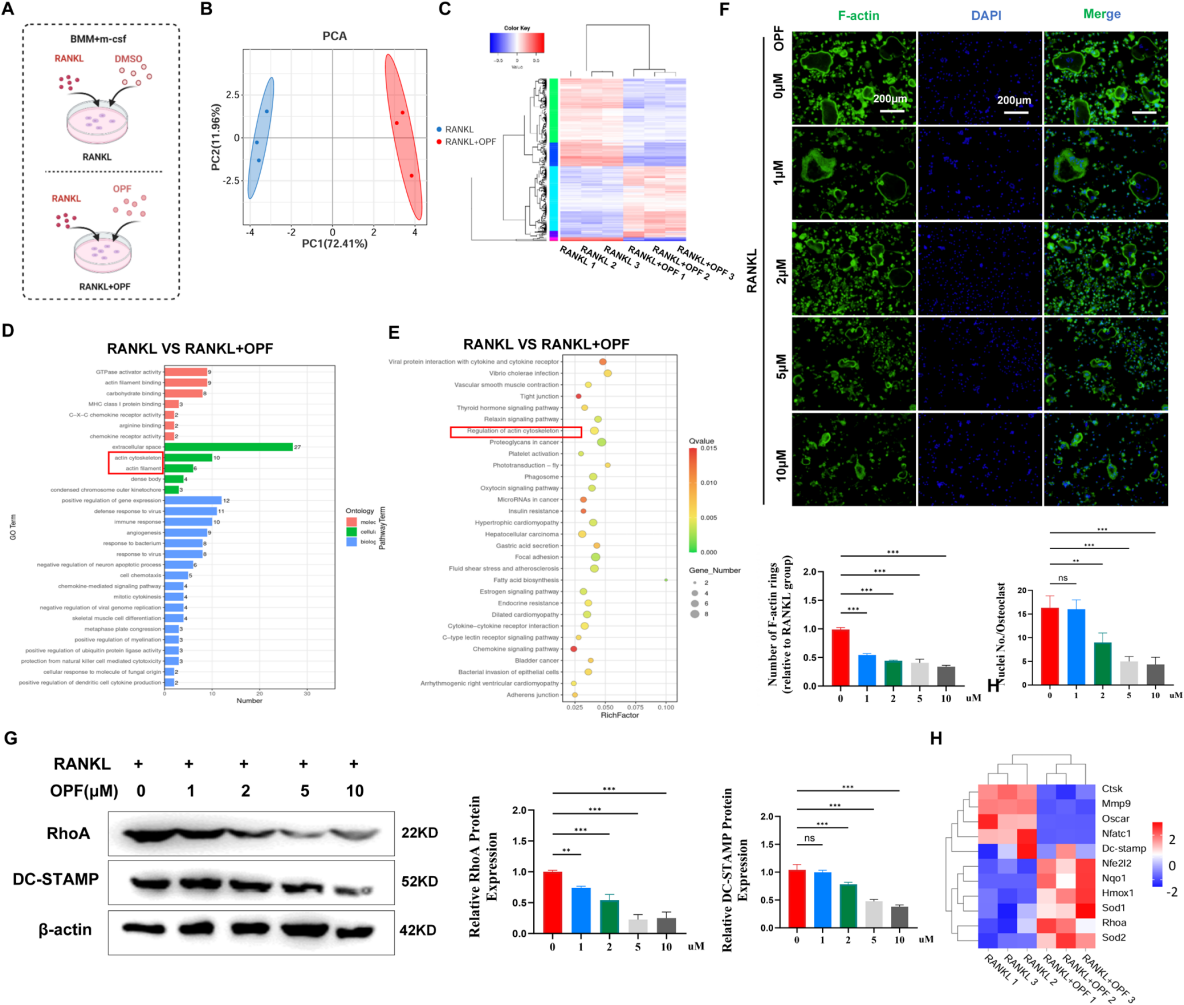
OPF involvement in regulating osteoclast cytoskeleton formation. (A) Schematic diagram of experimental groups. (B) PCA analysis of high‐throughput RNA sequencing data. (C) A heatmap reveals the differences in gene expression between the RANKL group and the RANKL+OPF group. (D) GO analysis of differentially expressed genes. (E) KEGG analysis of differentially expressed genes. (F) Immunofluorescence staining of F‐Actin (green) and DAPI (blue) in BMMs treated with RANKL and different concentrations of OPF. Scale bar = 200 μm. (G) Western blot results indicate the expression of RhoA and DC‐STAMP after OPF intervention at different concentrations during osteoclast differentiation, *n* = 3. ns, no significant difference; **p < 0.01; ***p < 0.001; relative to 0 μM group. (H) Heatmap showing the expression of osteoclast‐related and oxidative stress‐related genes in RANKL and RANKL+OPF groups.

To clarify how OPF suppresses osteoclast differentiation, we performed RNA sequencing in BMMs treated with RANKL in the presence or absence of OPF (Figure 3A). PCA showed tight clustering within each group and a clear separation between groups, supporting good reproducibility and overall sample consistency (Figure 3B). Differential expression analysis identified 251 differentially expressed genes (DEGs) between the RANKL and RANKL + OPF groups, including 136 upregulated and 115 downregulated genes (Figure 3C). Functional enrichment analyses further indicated that OPF‐responsive genes were prominently associated with osteoclast cytoskeleton organisation and related biological processes/pathways (Figure 3D,E). Given that cytoskeletal remodelling is essential for osteoclast spreading, fusion, and acquisition of bone‐resorptive capacity, these results suggested that OPF may inhibit osteoclastogenesis by disrupting cytoskeletal formation and maturation [30]. Consistently, F‐actin staining revealed that OPF markedly impaired actin ring/cytoskeletal organisation during osteoclast differentiation in a concentration‐dependent manner (Figure 3F). Quantitative analysis confirmed a significant reduction in F‐actin ring formation following OPF treatment (Figure 3F). To further connect the transcriptomic changes with key functional mediators, we examined representative genes involved in osteoclast differentiation and fusion. RhoA is a pivotal small GTPase governing actin cytoskeleton rearrangement and osteoclast maturation [31], while DC‐STAMP is indispensable for precursor fusion and multinucleated osteoclast formation [32]. Western blotting further demonstrated that OPF dose‐dependently reduced the protein levels of RhoA and DC‐STAMP during RANKL‐induced osteoclastogenesis (Figure 3G). Notably, OPF treatment was also associated with increased expression of oxidative stress–responsive genes, including Nfe2l2 (Nrf2), Nqo1, Hmox1, Sod1, and Sod2, alongside suppression of osteoclast‐related gene expression (Figure 3H). These transcriptomic changes point to a possible link between redox regulation and OPF‐induced impairment of osteoclast cytoskeletal organisation and maturation.

### 3.4 OPF Inhibits the Production of Intracellular ROS by Upregulating the Expression of Antioxidant Proteins

**FIGURE 4:**
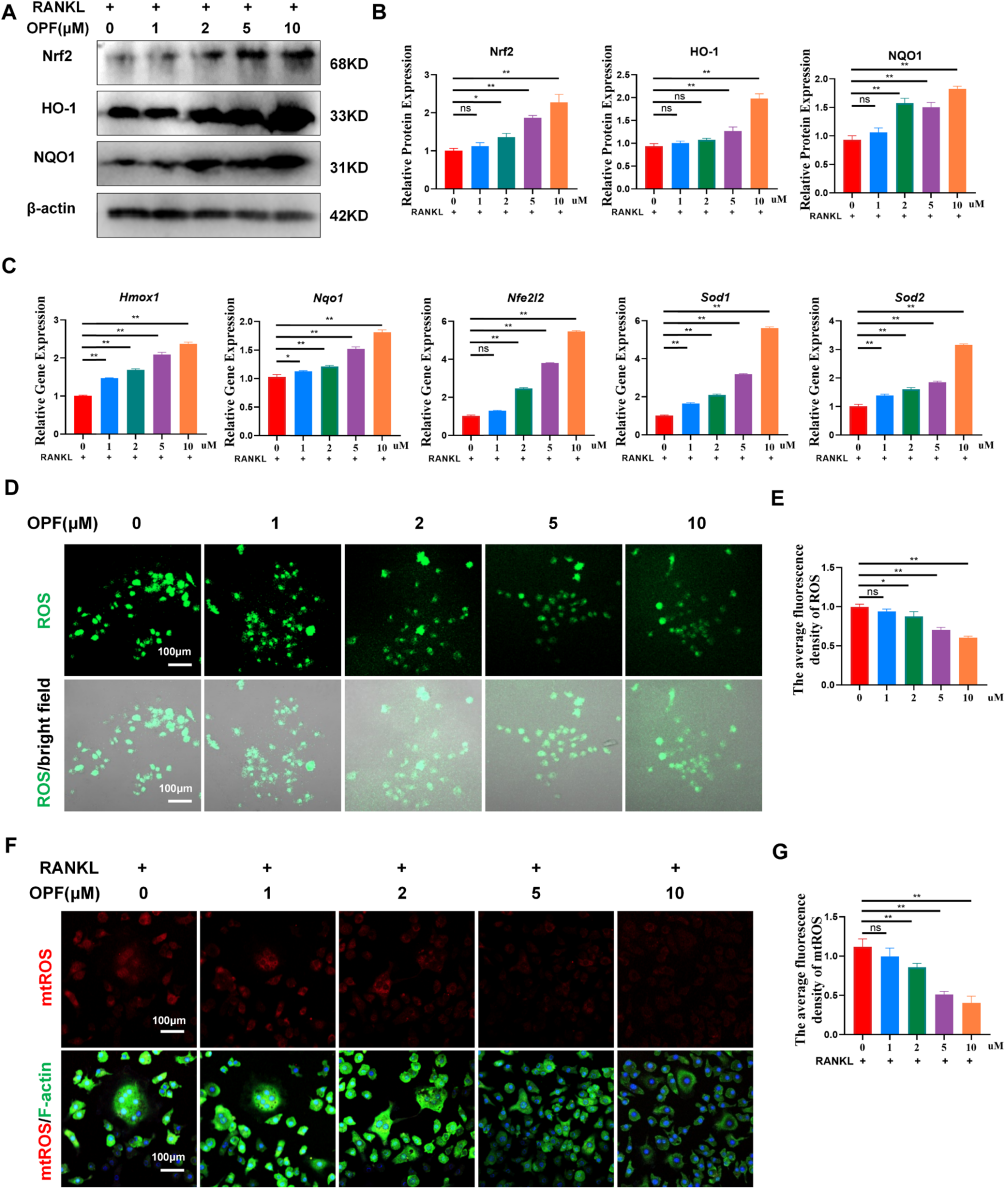
OPF inhibits the production of intracellular ROS by upregulating the expression of antioxidant proteins. (A) Western blot analysis revealed the effects of OPF intervention on the expression of Nrf2, HO‐1, and NQO1 in osteoclasts, *n* = 3. (B) Quantitative analysis of the proteins Nrf2, HO‐1, and NQO1 was performed, *n* = 3. (C) RT‐PCR demonstrated the impact of OPF intervention on the expression of antioxidant‐related genes *Nfe2l2*, *Hmox1*, *Nqo1*, *Sod1*, and *Sod2* in osteoclasts, *n* = 3. (D) Immunofluorescence staining showed the influence of a concentration gradient of OPF intervention on the generation of intracellular ROS, *n* = 3, Scale bar = 100 μm. (E) Flow cytometry revealed the effects of a concentration gradient of OPF intervention on the generation of intracellular ROS, *n* = 3. (F) Immunofluorescence staining indicated the effects of a concentration gradient of OPF intervention on the production of mtROS within cells, *n* = 3, Scale bar = 100 μm. (G) Flow cytometry demonstrated the impact of a concentration gradient of OPF intervention on the generation of mtROS in cells, *n* = 3. **p < 0.01.

Previous studies have demonstrated that OPF possesses strong antioxidant properties [33]. Furthermore, we investigated the regulatory effects of OPF on intracellular oxidative stress. Nrf2 is the primary transcription factor involved in the antioxidant response. When oxidative stress occurs within the cell, Nrf2 is released and translocated to the nucleus, where it binds to the antioxidant response element to initiate the expression of downstream antioxidant markers such as NQO1 and HO‐1 [34]. Our Western blot results indicate that a gradient concentration of OPF significantly promotes the expression of Nrf2, HO‐1, and NQO1 within the cells (Figure 4A,B). SOD1 and SOD2 are both members of the superoxide dismutase family, capable of catalysing the conversion of superoxide anions (O_2_^−^·) into hydrogen peroxide (H_2_O_2_) and oxygen (O_2_) [35]. Our RT‐PCR results show that OPF not only dose‐dependently enhances the expression of antioxidant‐related genes *Nfe2l2*, *Hmox1*, and *Nqo1*, but also promotes the production of *Sod1* and *Sod2* (Figure 4C). Furthermore, we explored the impact of OPF on intracellular ROS production. Results from immunofluorescence staining and flow cytometry suggest that a gradient concentration of OPF inhibits the generation of intracellular ROS (Figure 4D,E). Mitochondrial ROS are also a core driving factor of oxidative stress. Our study also found that OPF significantly inhibits the production of mtROS in osteoclasts (Figure 4F,G).

### 3.5 OPF Targets Nrf2 to Inhibit Osteoclast Cell Activation and Bone Resorption

**FIGURE 5:**
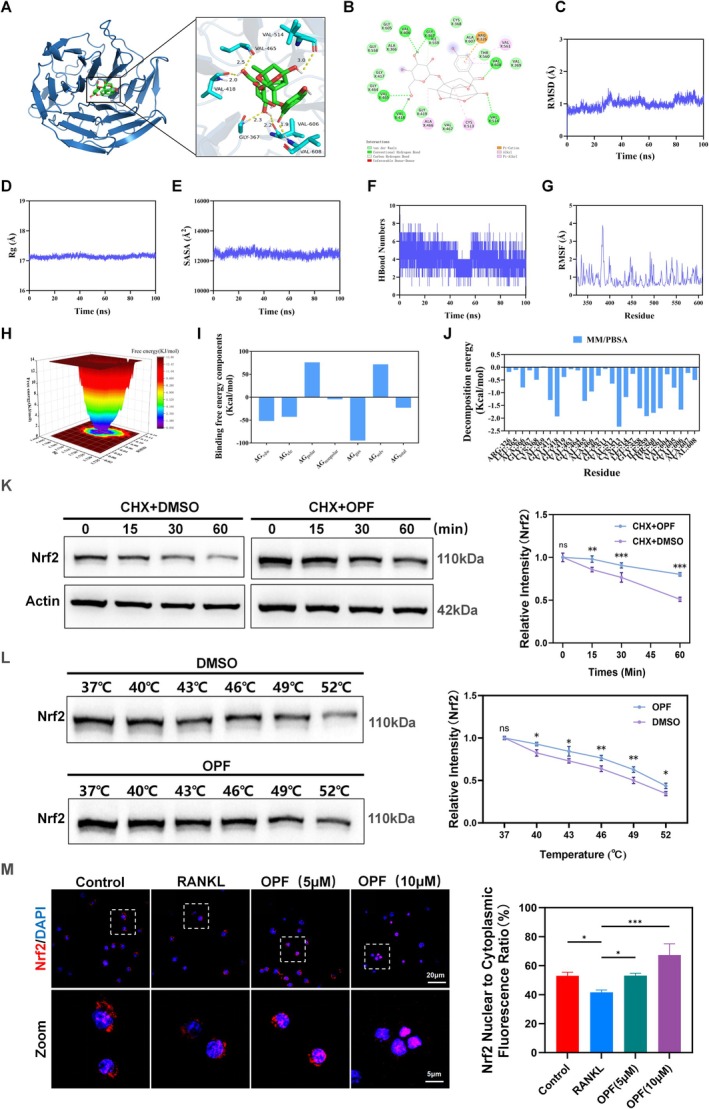
Molecular docking and molecular dynamics simulation of OPF with Nrf2. (A) Three‐dimensional docking model of OPF bound to Nrf2. (B) Two‐dimensional interaction diagram between OPF and Nrf2 residues. (C) Root mean square deviation (RMSD) of the Nrf2–OPF complex during the 100 ns molecular dynamics simulation. (D) Radius of gyration (Rg) of the Nrf2–OPF complex during the simulation. (E) Solvent‐accessible surface area (SASA) of the Nrf2–OPF complex during the simulation. (F) Number of hydrogen bonds between OPF and Nrf2 over simulation time. (G) Root mean square fluctuation (RMSF) of Nrf2 residues. (H) Free energy landscape of the Nrf2–OPF complex. (I) MM/PBSA binding free energy components of the Nrf2–OPF complex. (J) Per‐residue MM/PBSA energy decomposition of the Nrf2–OPF complex. (K) Nrf2 protein stability assessed by Cycloheximide (CHX) chase assay at indicated time points, with relative intensity quantified (right). (L) Cellular Thermal Shift Assay (CETSA) showing the thermal stabilization of Nrf2 by OPF across a temperature gradient (37°–52°C). (M) Representative immunofluorescence images showing the subcellular localization of Nrf2 (red) and DAPI (blue) in BMMs; The corresponding bar graph shows the ratio of nuclear to cytoplasmic fluorescence intensity. Data are presented as mean ± SD. *p < 0.05, **p < 0.001.

Nrf2 is a central regulator of cellular oxidative stress responses, and our previous results indicated that OPF markedly enhanced Nrf2 expression during osteoclast differentiation. To further explore whether OPF directly interacts with Nrf2, molecular docking and molecular dynamics (MD) simulations were performed. The docking results showed that OPF could be accommodated within the binding pocket of Nrf2 (Figure 5A), and the two‐dimensional interaction diagram revealed multiple interactions between OPF and surrounding residues of Nrf2 (Figure 5B). Specifically, OPF formed hydrogen bond interactions with GLY367, VAL418, VAL465, VAL514, VAL606, and VAL608 of Nrf2. In addition, hydrophobic interactions were observed between OPF and VAL561, CYS513, and ALA466, while extensive van der Waals interactions were formed with THR560, VAL369, ALA607, CYS468, ILE559, GLY605, ALA366, GLY558, GLY417, GLY464, GLY419, and VAL467. A π–cation interaction was also identified between OPF and ARG326, suggesting a stable binding mode between OPF and Nrf2. To further evaluate the stability of the Nrf2–OPF complex, MD simulations were conducted. The root mean square deviation (RMSD) analysis showed that the complex reached equilibrium and remained stable throughout the 100 ns simulation period (Figure 5C). Consistently, the radius of gyration (Rg) and solvent‐accessible surface area (SASA) exhibited minimal fluctuations during the simulation, indicating overall structural stability of the complex (Figure 5D,E). Hydrogen bond analysis demonstrated persistent interactions between OPF and Nrf2 over time (Figure 5F). Residue‐level flexibility analysis revealed limited fluctuations in most regions of Nrf2 upon OPF binding (Figure 5G). The free energy landscape further illustrated a dominant low‐energy conformational state of the complex (Figure 5H). Moreover, MM/PBSA analysis showed favourable binding free energy contributions, with key residues contributing to the interaction between OPF and Nrf2 (Figure 5I,J). To experimentally validate the direct physical interaction between OPF and Nrf2, a Cellular Thermal Shift Assay (CETSA) was performed. OPF treatment (10 μM) significantly increased the thermal stability of Nrf2 across a temperature gradient (37°–52°C) compared to the DMSO control, providing direct evidence of a physical OPF‐Nrf2 interaction in a cellular environment (Figure 5L). Given that Nrf2 activity is largely controlled by its degradation rate, we conducted a cycloheximide (CHX) chase assay to evaluate Nrf2 protein stability. OPF treatment markedly slowed Nrf2 degradation, extended its half‐life, and maintained higher Nrf2 protein levels than the CHX + DMSO group during the 60 min chase period (Figure 5K). Finally, we investigated whether the stabilization of Nrf2 led to increased nuclear activity. Immunofluorescence staining revealed that while RANKL stimulation decreased the nuclear presence of Nrf2, OPF treatment dose‐dependently reversed this effect. Quantitative analysis of the nuclear‐to‐cytoplasmic fluorescence ratio confirmed that OPF significantly promoted the nuclear translocation of Nrf2 (Figure 5M). In summary, these data demonstrate that OPF directly binds to Nrf2, protects it from degradation, and facilitates its translocation into the nucleus to activate the antioxidant response.

**FIGURE 6:**
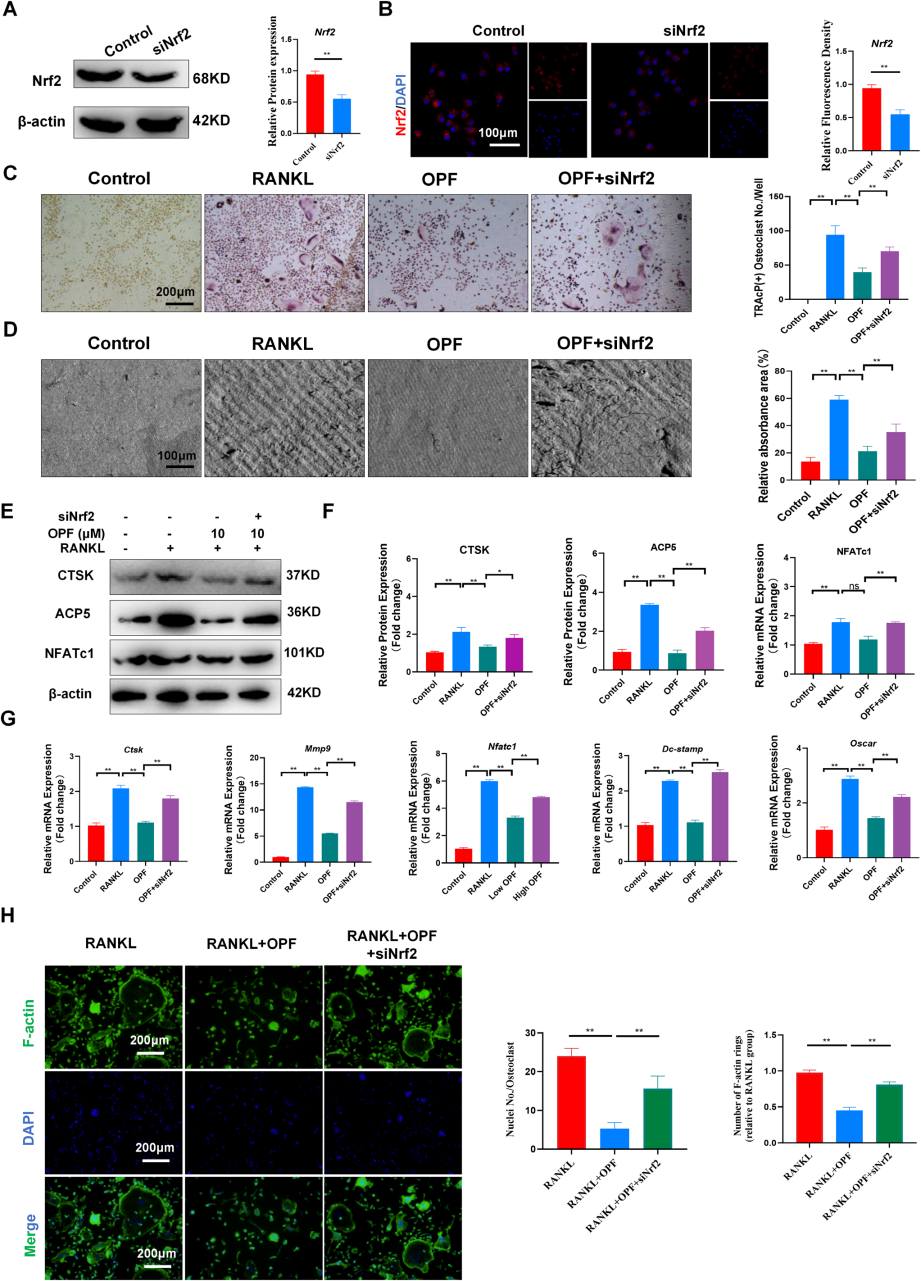
OPF targets Nrf2 to inhibit osteoclast cell activation and bone resorption. (A) Western blot reveals the intracellular Nrf2 levels after siNrf2 intervention, *n* = 3. (B) Immunofluorescence staining reveals the expression of Nrf2 in cells after siNrf2 intervention, *n* = 3, Scale bar = 100 μm. (C) TRAcP staining demonstrates the differentiation of osteoclasts following OPF combined with siNrf2 intervention, *n* = 3, Scale bar = 200 μm. (D) Bone plate resorption experiments reveal the level of bone resorption in osteoclasts after OPF combined with siNrf2 intervention, *n* = 3, Scale bar = 100 μm. (E) Western blot shows the expression of osteoclast‐related marker proteins CTSK, ACP5, and NFATc1, *n* = 3. (F) Quantitative analysis of the proteins CTSK, ACP5, and NFATc1, *n* = 3. (G) RT‐PCR reveals the expression levels of osteoclast‐related marker genes *Ctsk*, *Mmp9*, *Nfatc1*, *Dc‐stamp*, and *Oscar* following OPF combined with siNrf2 intervention. **p < 0.01. (H) Immunofluorescence staining of F‐Actin (green) and nuclei (DAPI, blue) in BMMs under different treatments (RANKL, RANKL+OPF, and RANKL+OPF + siNrf2). Scale bar = 200 μm.

To further interrogate the functional role of Nrf2 in OPF‐mediated inhibition of osteoclastogenesis, we designed siRNA targeting Nrf2 to achieve gene knockdown. Western blot analysis confirmed the efficiency of siNrf2, showing a marked reduction in Nrf2 protein levels in BMMs (Figure 6A), which was further validated by immunofluorescence staining (Figure 6B). We next applied siNrf2 during OPF‐treated osteoclast differentiation to assess whether Nrf2 depletion could reverse the inhibitory effects of OPF. While OPF treatment alone significantly suppressed osteoclast formation, Nrf2 knockdown partially restored osteoclast differentiation, as evidenced by increased numbers of mature osteoclasts (Figure 6C). Consistently, siNrf2 intervention also significantly enhanced osteoclast‐mediated bone resorption compared with OPF treatment alone (Figure 6D). At the molecular level, OPF treatment markedly downregulated the expression of key osteoclast marker proteins, including CTSK, ACP5, and NFATc1; however, these inhibitory effects were substantially attenuated upon Nrf2 knockdown (Figure 6E,F). In line with these protein‐level changes, siNrf2 significantly upregulated the transcription of osteoclast‐related genes (*Ctsk, Mmp9, Nfatc1, Dc‐stamp*, and *Oscar*) that had been suppressed by OPF (Figure 6G). Furthermore, F‐actin immunofluorescence staining demonstrated that Nrf2 knockdown promoted cytoskeletal organization and actin ring formation during osteoclast differentiation, counteracting the OPF‐induced impairment of osteoclast cytoskeleton assembly (Figure 6H). Collectively, these findings indicate that activation of the Nrf2 pathway is functionally involved in the inhibitory effects of OPF on osteoclast differentiation, cytoskeletal organization, and bone‐resorptive activity.

### 3.6 OPF Inhibits Oxidative Stress and Osteoclast Cytoskeleton Formation via Nrf2

**FIGURE 7:**
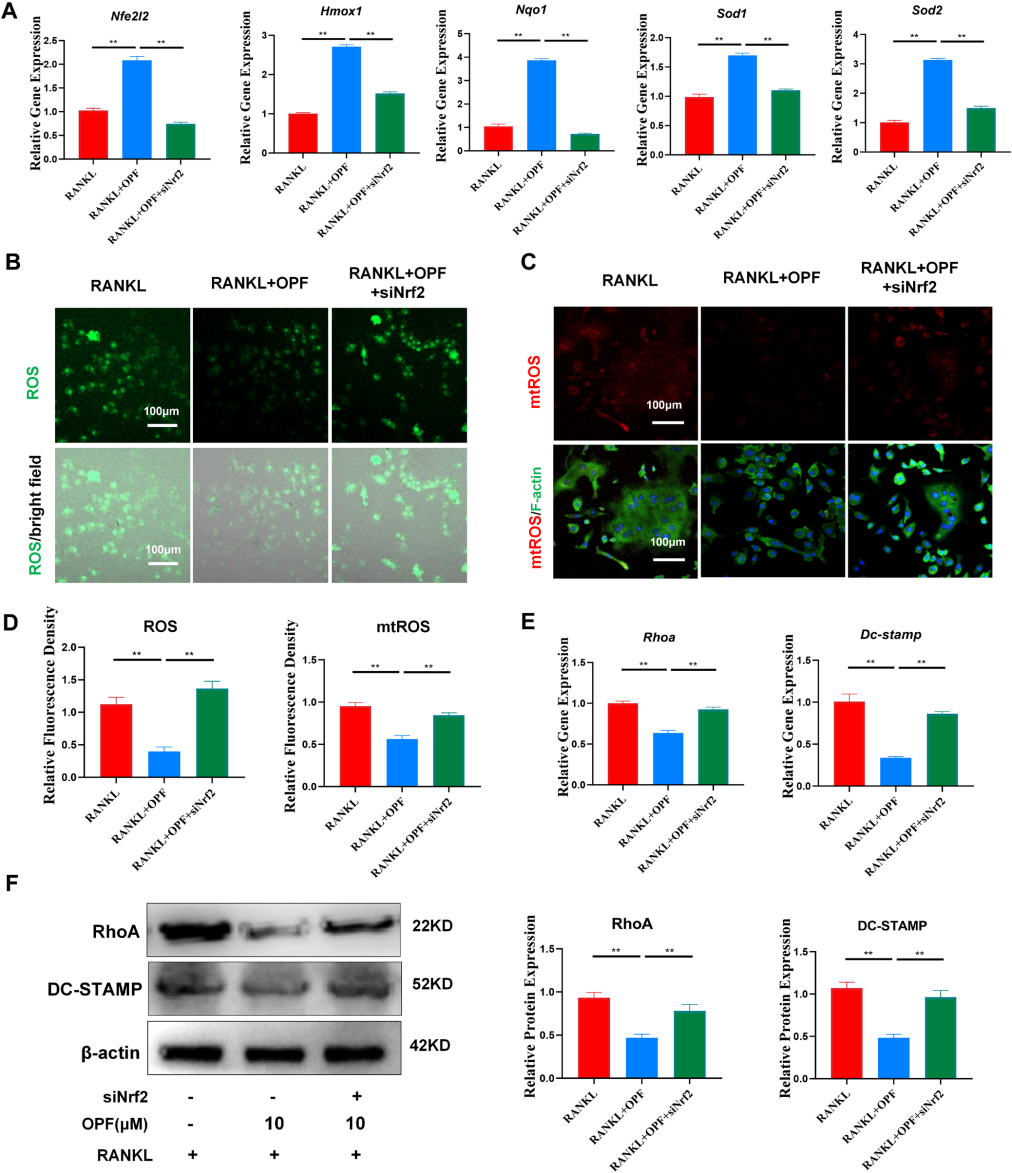
OPF inhibits oxidative stress and osteoclast cytoskeleton formation via Nrf2. (A) RT‐PCR revealed the expression of antioxidant‐related genes *Nfe2l2*, *Hmox1*, *Nqo1*, *Sod1*, and *Sod2* in osteoclasts, *n* = 3. (B) Immunofluorescence staining of ROS in osteoclasts, *n* = 3, Scale bar = 100 μm. (C) Immunofluorescence staining of mtROS in osteoclasts, *n* = 3, Scale bar = 100 μm. (D) Quantification of relative fluorescence density of ROS and mtROS. (E) RT‐PCR revealed the expression levels of RhoA and DC‐STAMP in osteoclasts after siNrf2 combined with OPF intervention, *n* = 3. (F) Western blot analysis showed the expression levels of RhoA and DC‐STAMP after siNrf2 combined with OPF intervention, *n* = 3. **p < 0.01; relative to RANKL+OPF group.

Subsequently, we again employed siNrf2 to intervene in the process of osteoclast differentiation mediated by OPF. We found that following OPF intervention, the expression of antioxidant‐related genes *Nfe2l2*, *Hmox1*, *Nqo1*, *Sod1*, and *Sod2* in osteoclasts significantly increased (Figure 7A). However, after the siNrf2 intervention, the expression levels of these related genes were markedly reversed. Furthermore, we observed that siNrf2 intervention led to an amplification in the levels of ROS and mtROS in osteoclasts (Figure 7B,D). Additionally, we discovered that following OPF intervention, the expression of cytoskeleton formation‐related markers RhoA and DC‐STAMP decreased, but after siNrf2 intervention, the expression of RhoA and Dc‐stamp significantly increased (Figure 7E,F).

### 3.7 OPF Inhibits Titanium Particle‐Induced Osteolysis and Osteoclast Activation in Mouse Calvaria

**FIGURE 8:**
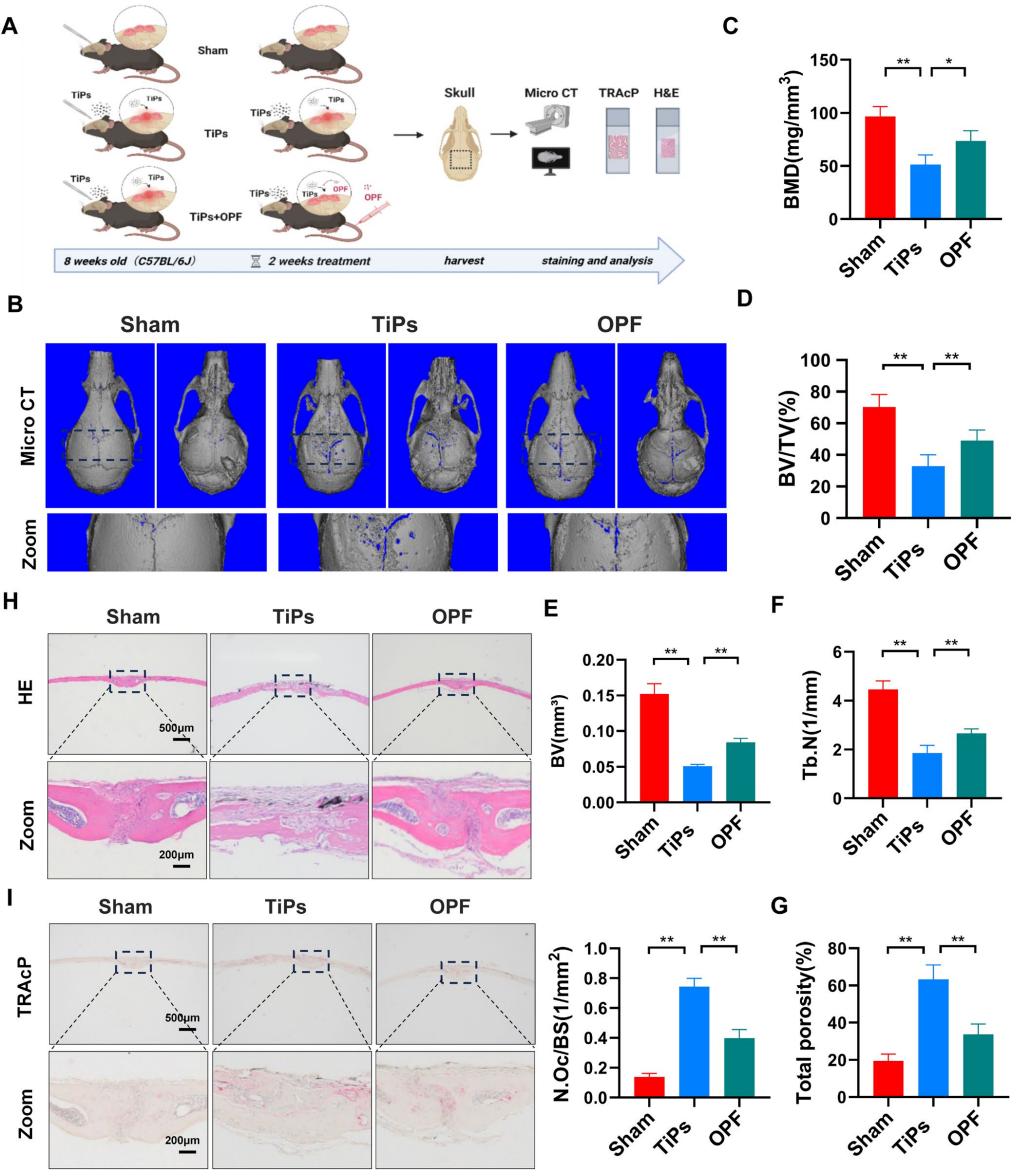
OPF inhibits titanium particle‐induced osteolysis and osteoclast activation in mouse calvaria. (A) A schematic diagram of OPF intervention for the treatment of bone dissolution. (B) Micro‐CT reveals three‐dimensional images of mouse cranial bones after OPF intervention, *n* = 7. (C‐G) Analysis of cranial bone parameters in mice, *n* = 7. (H) H&E staining of mouse cranial bones, *n* = 7, Scale bar = 200 μm. (I) TRAcP staining of mouse cranial bones and quantitative analysis of the number of osteoclasts relative to the TiPs group. *n* = 7, Scale bar = 200 μm.

We developed a mouse calvarial osteolysis model. The study included three modelling groups: a sham surgery group, a titanium particle group, and a titanium particle combined with OPF group (Figure 8A). Micro‐CT analysis revealed significant calvarial erosion in the titanium particle group relative to the sham surgery group. In contrast, the erosion in the titanium particle + OPF group was significantly reduced (Figure 8B). Quantitative analysis showed that titanium particle intervention reduced local bone density, bone volume fraction, bone volume, and trabecular number while increasing porosity. OPF treatment enhanced bone density and alleviated alterations in bone parameters induced by titanium particles (Figure 8C,G). Histological staining of calvarial sections corroborated the therapeutic impact of OPF on osteolysis induced by titanium particles. H&E staining indicated significant erosion and soft tissue proliferation in the titanium particle group, which were notably reduced by OPF treatment (Figure 8H). TRAcP staining was conducted on calvarial sections to examine the impact of OPF treatment on osteoclastogenesis induced by titanium particles (Figure 8I). These results indicated that OPF treatment significantly reduced the TRAcP‐positive osteoclasts induced by titanium particles.

## 4 Discussion

TJA is a widely used surgical method for treating degenerative and post‐traumatic severe arthritis of the hip and knee joints [36, 37]. However, aseptic loosening caused by wear debris from the prosthesis, along with subsequent bone resorption, may lead to TJA failure and necessitate surgical revision [38, 39]. Nanoparticles generated from prosthetic wear accumulate at the bone‐implant interface and are phagocytosed by immune cells, triggering an inflammatory response that further promotes osteoclast‐mediated bone resorption, ultimately resulting in the onset of periprosthetic osteolysis [40].

Based on the potential pathological mechanisms of PPO, efforts have been made to utilize bone resorption inhibitors for its treatment [41]. However, the therapeutic effects remain unsatisfactory and are often accompanied by the occurrence of side effects. Interventions targeting osteoclast function can mitigate the pathological process of PPO and extend the longevity of the prosthesis by improving specific mechanisms. Therefore, it is necessary to further explore effective strategies that can inhibit osteoclast activation in order to treat wear particle‐induced bone resorption [42, 43].

In the process of modernization, traditional Chinese medicine will provide unique solutions for human health. Paeoniflorin is a natural monoterpene glycoside extracted from plants of the Paeoniaceae family and is one of the core active components of the traditional Chinese medicine Shaoyao [44]. Previous studies have shown that Paeoniflorin possesses multiple effects, including anti‐inflammatory, antioxidant, and immunoregulatory properties [45]. Oxypaeoniflorin is a derivative of Paeoniflorin, the core active ingredient of Shaoyao. Currently, Oxypaeoniflorin is still in the early stages of research, but recent studies have indicated that it has positive therapeutic effects in myocardial ischemia/reperfusion injury and acute lung injury [46]. Previous research revealed that Paeoniflorin improves collagen‐induced arthritis by inhibiting the nuclear factor‐κB signalling pathway involved in osteoclast differentiation [47]. However, it remains unclear whether Oxypaeoniflorin can regulate osteoclast differentiation and be used to treat particle‐induced bone resorption.

In our study, we found that OPF inhibits osteoclast activation and bone resorption in a dose‐dependent manner. Furthermore, our results also suggest that OPF significantly downregulates the expression of osteoclast‐related markers. Through transcriptome sequencing, we discovered that OPF primarily affects the cytoskeletal formation of osteoclasts, and a concentration gradient of OPF significantly downregulates the expression of cytoskeletal formation‐related proteins RhoA and DC‐STAMP.

Previously, Ren et al. discovered that paeoniflorin alleviates AngII‐induced cardiac hypertrophy in H9c2 cells by regulating oxidative stress and the Nrf2 signalling pathway [22]. Zhou et al. found that paeoniflorin inhibits oxidative stress and NLRP3 inflammasome‐mediated pyroptosis by activating the Nrf2 signalling pathway, thereby mitigating acute lung injury induced by particulate matter [48]. Nevertheless, recent studies have also indicated that oxypaeoniflorin exhibits excellent antioxidant activity against cell injury [49]. Subsequently, in our study, we also found that OPF can upregulate Nrf2 activity in osteoclasts and further promote the expression of intracellular antioxidant‐related genes *Hmox1*, *Nqo1*, *Sod1*, and *Sod2*. In the molecular mechanisms regulating osteoclast activation, the generation and metabolic imbalance of ROS constitute the core driving force of the pathological process [50]. Mitochondria serve as the primary site for intracellular ROS production. In our study, we confirmed that OPF can inhibit the production of intracellular ROS and mtROS.

Nrf2, as an important transcription factor regulating oxidative stress, plays a crucial role in inducing the body's antioxidant response [51]. Studies have shown that during the differentiation of osteoclasts stimulated by RANKL, the negative regulator of Nrf2, Keap1, is upregulated, which lowers the Nrf2/Keap1 ratio and downregulates cytoprotective enzymes [52]. In our study, we constructed small interfering RNA targeting Nrf2 knockdown. We found that after siNrf2 intervention, the expression of osteoclast antioxidant‐related genes was significantly downregulated, and osteoclast activation was further amplified, with a significant increase in cytoskeletal formation. Previously, numerous molecular drugs have been confirmed to inhibit osteoclast differentiation and treat bone loss by activating Nrf2. RTA‐408 is a triterpenoid compound currently under clinical investigation, and studies have shown that RTA‐408 can antagonize the interaction between STING and the E3 ubiquitin ligase TRAF6, thereby weakening the endogenous NF‐κB signalling required for osteoclast differentiation [53]. Metastasis‐associated protein 1 (MTA1), belonging to the metastasis‐associated protein family, is an important component of the intracellular histone deacetylase complex. Recent research indicates that the absence of MTA1 reduces the basal expression of Nrf2 and inhibits the expression of Nrf2‐mediated antioxidant enzymes, leading to enhanced osteoclast formation [54]. Our study reveals that OPF mainly mediates osteoclast activation in vitro by targeting the Nrf2 signalling pathway.

In the in vivo part of this study, we employed a titanium particle‐induced calvarial osteolysis model and found that OPF significantly alleviated bone erosion and reduced osteoclast formation. The murine calvarial model remains one of the most commonly used platforms for primary in vivo investigation of particle‐induced osteolysis because of its technical simplicity, reproducibility, and suitability for mechanistic and proof‐of‐concept therapeutic studies [12, 55]. In this context, our use of local subperiosteal delivery was intended to increase drug exposure at the particle‐challenged site while minimizing potential systemic interference. Notably, local delivery strategies have also been adopted in previous calvarial osteolysis studies, supporting the rationale for site‐directed intervention in this model [56, 57]. At the same time, it should be recognized that the classical calvarial model mainly reflects acute particle‐driven inflammatory bone loss and does not fully recapitulate the chronic implant‐bone interface, load‐bearing mechanical environment, or prolonged generation of heterogeneous wear debris observed in clinical PPO [58]. Therefore, the present findings should be interpreted as proof‐of‐concept evidence supporting the local anti‐osteolytic activity of OPF. Further validation in more clinically relevant implant‐associated or progressive particle‐delivery models will be important to better define its translational potential.

## 5 Conclusion

In this study, we explored the role of OPF in the differentiation of osteoclasts and titanium particle‐induced bone resorption. In vitro experiments revealed that OPF significantly inhibits osteoclast activation and bone resorption and is involved in the suppression of osteoclast cytoskeletal formation. Mechanistically, OPF may target Nrf2 to promote the generation of antioxidant‐related genes in osteoclasts and the clearance of ROS. These findings suggest that OPF may be a promising therapeutic approach for treating periprosthetic bone resorption and preventing aseptic loosening.

## Author Contributions

**Feng Zhu:** conceptualization, methodology, software, writing – original draft. **Huaqiang Tao:** data curation, writing – original draft. **Kai Chen:** data curation, writing – original draft. **Wenming Li:** data curation, writing – original draft. **Zhidong Wang:** software, validation. **Yaozeng Xu:** software, validation. **Dechun Geng:** supervision, funding acquisition, project administration. **Zebin Wu:** visualization, investigation, writing – original draft. **Qihan Wang:** software. **Shujun Lv:** writing – review and editing, resources. **Jinxuan Hei:** writing – review and editing, methodology, formal analysis, investigation, visualization.

## Funding

This work was supported by Jiangsu Medical Research Project, ZD2022014. Program of Suzhou Health Commission, GSWS2022002. National and Local Engineering Laboratory of New Functional Polymer Materials, SDGC2205. Foundation of National Center for Translational Medicine (Shanghai) SHU Branch, SUITM‐202403. Project of MOE Key Laboratory of Geriatric Diseases and Immunology, KJS2502. Priority Academic Program Development of Jiangsu Higher Education Institutions, PAPD.

## Conflicts of Interest

The authors declare no conflicts of interest.

## Data Availability

The data that support the findings of this study are available from the corresponding author upon reasonable request.
